# Hsa-miR-574-5p negatively regulates MACC-1 expression to suppress colorectal cancer liver metastasis

**DOI:** 10.1186/1475-2867-14-47

**Published:** 2014-06-07

**Authors:** Zhe Cui, Jian Tang, Jinxian Chen, Zheng Wang

**Affiliations:** 1 Department of General Surgery, Ren Ji Hospital, School of Medicine, Shanghai Jiao Tong University, Shanghai, China

**Keywords:** Colorectal cancer, MACC-1, miRNA, Liver metastasis

## Abstract

**Objective:**

The aim of this study was to investigate the relationship of MACC-1 (metastasis-associated in colon cancer 1) and microRNA (miRNA) hsa-miR-574-5p and the function of hsa-miR-574-5p in colorectal cancer liver metastasis.

**Methods:**

Liver-metastatic nude mice model was constructed by injecting two human colorectal cancer cell lines (SW1116 and HCT116) labeled with green fluorescent protein (GFP) through spleen, and liver metastasis incidences were evaluated. We identified miRNAs that might regulate MACC-1 expression by bioinformatics analysis and further investigated the relationship of MACC-1 and hsa-miR-574-5p by luciferase reporter assay, quantitative RT-PCR and western blot. The effect of hsa-miR-574-5p on colony formation, cell invasion and cell spheroid formation was investigated by antisense transfected HCT116 cells and miRNA mimic transfected SW1116 cells.

**Results:**

The volume of liver metastasis induced by SW1116 cells (25.0 ± 4.4%) was significantly higher than that induced by HCT116 cells. Bioinformatics analysis showed hsa-miR-574-5p negatively regulated MACC-1 and then their interaction was demonstrated at mRNA and protein level. The direct relation between them was confirmed by luciferase reporter assay. And the knockdown of has-miR-574-5p demonstrated increased colony formation, cell invasion and cell spheroid formation in HCT116 cells, compared to control group (P < 0.05). Reverse results were obtained in mimic transfected SW1116 cells.

**Conclusion:**

Our work firstly demonstrated that hsa-miR-574-5p negatively regulated MACC-1 expression in colorectal cancer cells. It was partly elucidated that hsa-miR-574-5p played a suppressive role in colorectal cancer liver metastasis by negatively directing MACC-1 expression, offering a novel therapeutic approach for colorectal cancer liver metastasis.

## Introduction

Colorectal cancer liver metastasis is a bottleneck worldwide, which restricts patients from long-term survival. There are about 500,000 cases of colorectal cancer liver metastasis every year [1]. Once liver metastasis happens, the prognosis is often very poor [2]. Until recently, the incidence of liver metastasis for colorectal cancer patients is still very high. More than 70% patients with liver metastasis cannot be surgically resected [2]. Even after hepatectomy, the 2-year recurrence rate is as high as 75%, and 5-year overall survival rate is only 26.8% [3,4]. Although high attention has been paid to the colorectal cancer liver metastasis in clinical practice, the underlying molecular mechanism is still limited, thus lacking of effective prevention and intervention. Therefore, it is imperative to explore the mechanisms and look for new intervention targets and methods for colorectal cancer liver metastasis in both clinical practice and basic research.

It is a dynamic process for cancer cells to spread from the primary tumor to distant organs, and to develop metastasis. More than one gene are involved in this continuous and multiple-stage process, involving many aspects such as tumor cell adhesion, cell invasion, extracellular matrix remodeling, angiogenesis, genesis of lymphatic vessels, and immune system [5]. Recent study has shown that tumor metastasis is closely related to cancer cell epithelial-mesenchymal transition (EMT) and cancer stem cells (CSC) [6]. The high expression of α, β integrin in colorectal cancer cells facilitates the integration with portal and hepatic venous epithelial cells and the homing [7,8]. Metastasis-associated in colon cancer 1 (MACC-1) is recently discovered as an extremely important metastasis-related gene to regulate the metastasis of colorectal cancer as well as a variety of other solid tumors [9]. MACC-1, which is located on chromosome 7 (7p21.1), regulates HGF/c-Met signaling pathway that is involved in three major aspects of cancer metastasis-EMT, homing and CSC [10-13]. MACC-1 could be targeted by miR-143 to inhibit cell invasion and migration in colorectal cancer [14]. It has been reported that down-regulation of miR-1 and increase of MACC1 contributed to overexpression of Met in human colon cancer [15].

Lots of evidences have shown that microRNAs (miRNAs), the important regulators of functional genes, are involved in oncogenesis and tumor development [16,17]. Existing data also support that miRNAs expression are abnormal in the colorectal cancer cells. For example, low expression of miR-145 is related to the colorectal cancer [18], yet also reported in lung cancer [19] as CRC and lung cancer share epidermal growth factor receptor (EGFR) property [18,19]. The inflammatory cytokines such as IL (interleukin)-6, IL-8, IL-10, IL-12a and NOS2a (nitric oxide synthase), are positively correlated to miR-21 expression in colorectal cancer adjacent tissues, both of which are independent contributors to the poor prognosis of colorectal cancer [20]. It has been reported that hsa-miR-574-5p decreased CBR1 (carbonyl resuctase 1) gene expression and activity [21] and negatively regulated Qki6/7 to impact β-catenin/Wnt signalling and the development of colorectal cancer [22]. However, it is not completely clear which miRNAs are the regulators for MACC-1 which is the key candidate gene for colorectal cancer liver metastasis. Therefore, it is necessary to investigate the effects and molecular mechanisms of MACC-1 and its upstream regulatory miRNAs to modulate colorectal cancer liver metastasis.

In the present work, we identified the miRNAs profiles that might regulate the MACC-1 expression by bioinformatics analysis and investigated the relation between miRNA and MACC-1 expression. Then the function role of miRNA in colorectal cancer liver metastasis was further investigated by experiment design.

## Materials and methods

### Animals

Four-week-old male athymic BALB/c nu/nu nude mice (weighing from 15 to 18 g) were obtained from Shanghai SLAC Laboratory Animal Co. Ltd. (China). The bedding materials, drinking water, feed pellets and other items in contact with the animals were all autoclaved. The experiment and feedings were in SPF (specific-pathogen-free) condition according to the SYXK 2007–0001 standard. The animals were cultured in the animal lab in Shanghai Medical College of Fudan University in accordance with Guide for the Care and Use of Laboratory Animals. This study was approved by Ethics Committee of the Renji Hospital, Shanghai Jiaotong University School of Medicine, Shanghai, China.

### Cell culture and infection

Human colorectal cancer cell lines SW1116 and HCT116 were provided by Shanghai Institute of Digestive Disease. Cells were grown in complete medium supplemented with 10% fetal bovine serum (Gibco, CA, USA) and cultured in a 37°C humidified atmosphere of 5% CO_2_. One milliliter volume of pCDH cDNA lentivectors/green fluorescent protein (GFP) (5 × 10^8^ TU/mL, Shanghai Ming Hong Biotech Co. Ltd., China) was added to 1 ml single cell suspension (1 × 10^7^/mL cells) of SW1116 and HCT116 to infect cells (multiplicity of infection was 50). The lentivirus infection efficiency was observed using fluorescence microscope 3 days after infection. Cells were collected 5 days after infection for subsequent animal model development.

### Liver-metastatic model construction

Prior to trial, 40 nude mice were fasted for 12 h and then weighted. The mice were anesthetized with an intraperitoneal injection of 1% pentobarbital sodium (45 mg/kg) and placed in a supine position on the operating table. The skin was disinfected with 75% alcohol and oblique incision was made on left back (below conjunction of left posterior axillary line and costal margin) for 0.5 to 1.0 cm to expose spleen. Then the spleen was pulled out of the abdominal cavity gently and 5 × 10^6^/mL colorectal cancer cells in single suspension were injected slowly into spleen using five-gauge needle. Each nude mouse was injected for 0.2 ml over 3 min with visible splenic capsule swelling and whitening. After injection and needle withdrawal, 75% ethanol cotton swab was used for hemostasis oppression for 2 min, by which extravasated cancer cells were also killed to prevent intra-abdominal metastasis. The spleen was removed at 10 min after injection and then 3–0 absorbable sutures were used for abdomen closure. The mice were fed normal diet after waking up from anesthesia. The whole operation process followed the principles of aseptic surgery. Thirty-five days later after injection, the mice were sacrificed. The distribution of colorectal cancer cells in various organs were observed with fluorescence dissecting microscope (Qwin, Leica). The percentage of fluorescent area to the liver surface area was used to reflect liver metastasis and calculate the incidence.

### Bioinformatics analysis of miRNAs that regulate the MACC-1 expression

We extracted the whole genome sequence of MACC-1 from the National Center for Biotechnology Information (NCBI, Gene ID: 346389), especially the 3′-UTR (untranslated regions) sequence. The miRNAs profile that might regulate MACC-1 expression was predicted using miRbase ( http://www.mirbase.org/index.shtml) and microRNA.org ( http://www.microrna.org/microrna/home.do). Expect (E) value was used to choose the probable regulation miRNA. The smaller the E value is, the more probable the miRNA regulation of MACC-1 is.

### RNA extraction and quantitative RT-PCR (qRT-PCR) analysis

Total RNA was extracted from colorectal cancer cell lines (SW1116 and HCT116) with TRIzol reagent (Invitrogen, CA, USA). Housekeeping gene of glyceraldehyde-3-phosphate dehydrogenase (GAPDH) was used as internal control. MACC-1 mRNA level was measured with Access RT-PCR kit (Promega, USA). Hairpin-it miRNAs qPCR kit (GenePharma, China) was used to detect the hsa-miR-574-5p expression in SW1116 and HCT116 cell lines with stem-loop reverse transcription. The primers used in the study were shown as follows: MACC-1 (3′UTR sequence) primers, forward 5′-TTGCGGAGGTCACCATAGC-3′; reverse 5′-TTTCCAACAACGGGCTCA-3′; GAPDH primers, forward 5′-GGGCTGCTTTTAACTCTG-3′; reverse 5′-TGGCAGGTTTTTCTAGACGG-3′.

### Luciferase reporter assay

The amplified PCR products of 3′UTR sequence of wild type MACC-1 or mutant MACC-1 containing the putative has-miR-574-5p binding site were transformed into 293 T cells by pGL3 vector. The procedures were performed according to manufacture’s instruction of lipofectamine 2000 (Invitrogen, USA). At 24 h after transfection, luciferase activity was detected by Dual-Glo™ Luciferase Assay System (E2920, promega) and normalized by Renilla activity.

### Transfection of hsa-miR-574-5p antisense or miRNA mimic into SW1116 and HCT116 cell lines

Lipofectamine 2000 was used for oligonucleotide transfection. Three transfection groups were used for each cell line: control group which was transfected with 5 mg/L liposome only; mimic oligonucleotide group (mimic group) which was transfected with mixture of 100 nM mimic oligonucleotide and liposome; antisense oligonucleotide group (inhibitor group) which was transfected with mixture of 100 nM antisense oligonucleotide and liposome. The transfection was conducted in triplicate for each group. Cells were observed by inverted microscopy 24, 48, 72, and 96 h after transfection. Oligonucleotides expression was confirmed by RT-PCR.

### Western-blot to detect MACC-1 protein expression in hsa-miR-574-5p antisense or miRNA mimic transfected SW1116 and HCT116 cell lines

The cells were lysed with 2× lysis buffer on ice for 15 min and broken by ultrasonic disrupter at 4°C. After centrifugation at 12,000 g for 15 min, protein concentration in supernatant was measured. Protein (40 μg) was loaded onto a 10% SDS-PAGE gel (30 mA for 2 h) and transferred onto PVDF membrane (400 mA for 2 h, at 4°C). After probed with 1:1000 diluted rabbit polyclonal MACC-1 antibody (Abcam, MA, USA) at 4°C overnight, the blots were subsequently incubated with HRP (horseradish peroxidase) - conjugated secondary antibody (1:5000). The same results were repeated for seven times.

### Cell colony formation, invasion and spheroid formation after transfection

Log phase cells were digested by trypsin and re-suspended in complete medium to prepare cell suspension. Hemacytometer was used to determine cell count. Cells were seeded at a density of 500 cells per well into 96-well plates in triplicate for each group, and incubated for another 3 days. Medium was changed every 3 days. Cellomics Array Scan was used to scan and image each well to analyze number and size of clones as well as the number of cells in each clone.

For invasion assay, 1.0 × 10^5^ cells in 1% FBS (fetal bovine serum) were added to upper chamber pre-coated with 80 μL diluted matrigel matrix (BD, NJ, USA). Then 600 μl complete medium was added to the matched lower chamber. After 24–72 h incubation, noninvaded cells were removed from the upper surface of the transwell membrane with a cotton swab. Invaded cells on the lower membrane surface were fixed in paraformaldehyde for 15 min, and stained with 500 μL Giemsa solution. Five randomly selected fields were photographed with Image-Pro Plus software, and then counted.

Cell suspension (5 × 10^3^ cells, 200 μl) was added into each well of a 96-well plate. Relatively loose spheroids were visible after 3 days. The medium was then refreshed and cells were cultured for another 3 days to form bigger spheroids. Inverted phase contrast microscope was used to observe the size and morphology of cell spheroids. The diameter of spheroid was measured at day 7 and the size was compared among experimental groups [23].

### Statistical analysis

All data were expressed as mean ± standard deviation (SD) and processed using SPSS12.0 statistical software. A P-value less than 0.05 was considered to be statistically significant.

## Results

### Bioinformatics analysis of miRNAs regulating MACC-1 expression

The miRNAs that might regulate the MACC-1 gene expression was identified by bioinformatics analysis. The top 15 were shown in Table 1. Among those miRNAs, hsa-miR-574-5p expression level may be correlated to MACC-1 expression for the E value (0.10) was relatively lower.

**Table 1:** The miRNAs profile that might regulate the MACC-1 gene expression (Top 15)

**Accession**	**ID**	**Query start**	**Query end**	**Subject start**	**Subject end**	**strand**	**score**	**E (expect) value**	**Alignment**
MIMAT0015090	hsa-miR-1273d	21	45	1	25	-	98	0.015	Align
MIMAT0004795	hsa-miR-574-5p	81	103	1	23	+	88	0.10	Align
MIMAT0018079	hsa-miR-1273e	30	47	5	22	-	81	0.39	Align
MIMAT0005926	hsa-miR-1273	863	886	1	24	-	75	1.1	Align
MIMAT0005876	hsa-miR-1285	870	889	1	20	-	73	1.7	Align
MIMAT0003284	hsa-miR-616*	89	108	3	22	-	73	1.8	Align
MIMAT0002820	hsa-miR-497	371	390	2	21	-	73	1.8	Align
MIMAT0015090	hsa-miR-1273d	243	267	1	25	+	71	2.5	Align
MIMAT0004952	hsa-miR-665	216	229	3	16	+	70	3.0	Align
MIMAT0005583	hsa-miR-1228	189	205	4	20	+	67	5.7	Align
MIMAT0005577	hsa-miR-1226	88	104	6	22	+	67	3.2	Align
MIMAT0004805	hsa-miR-616	91	105	2	16	+	66	6.9	Align
MIMAT0003239	hsa-miR-574-3p	49	70	1	22	-	65	8.4	Align
MIMAT0005944	hsa-miR-1252	112	133	1	22	-	65	8.4	Align
MIMAT0009979	hsa-miR-2054	250	271	2	23	-	65	8.4	Align

### Incidences of liver metastasis induced by HCT116 or SW1116 tumor cells

**Figure 1:**
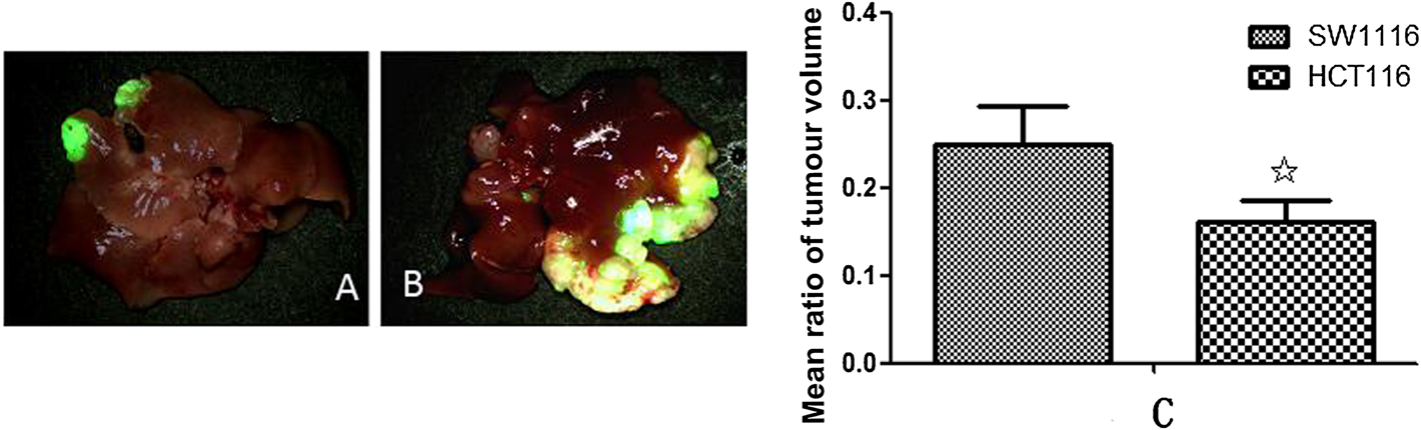
**Comparison of liver metastasis incidence in SW1116 and HCT116 injected nude mice. A**. liver metastasis in HCT116 cells injected nude mice; **B**. liver metastasis in SW1116 cells injected nude mice; **C**. SW1116 cells shows higher incidence of liver metastasis than HCT116 cells (^✩^P < 0.01 *vs.* SW1116).

Two human colorectal cancer cell lines SW1116 and HCT116 labeled with GFP were injected through spleen into male athymic BALB/c nu/nu nude mice to develop liver-metastatic models, respectively and liver-metastatic mice model was successfully constructed. Five weeks after spleen injection, GFP expression was visible in livers from 7 out of 20 nude mice in SW1116 group (7/20). While it was visible in livers only from 6 out of 20 nude mice in HCT116 group (6/20). The mean ratio of tumor volume induced by SW1116 cells (25.0 ± 4.4%) was significantly higher than that induced by HCT116 cells (16.0 ± 2.5%, P < 0.01, Figure 1A, B, C).

### MACC-1 and hsa-miR-574-5p mRNA levels in SW1116 and HCT116 cell lines

**Figure 2:**
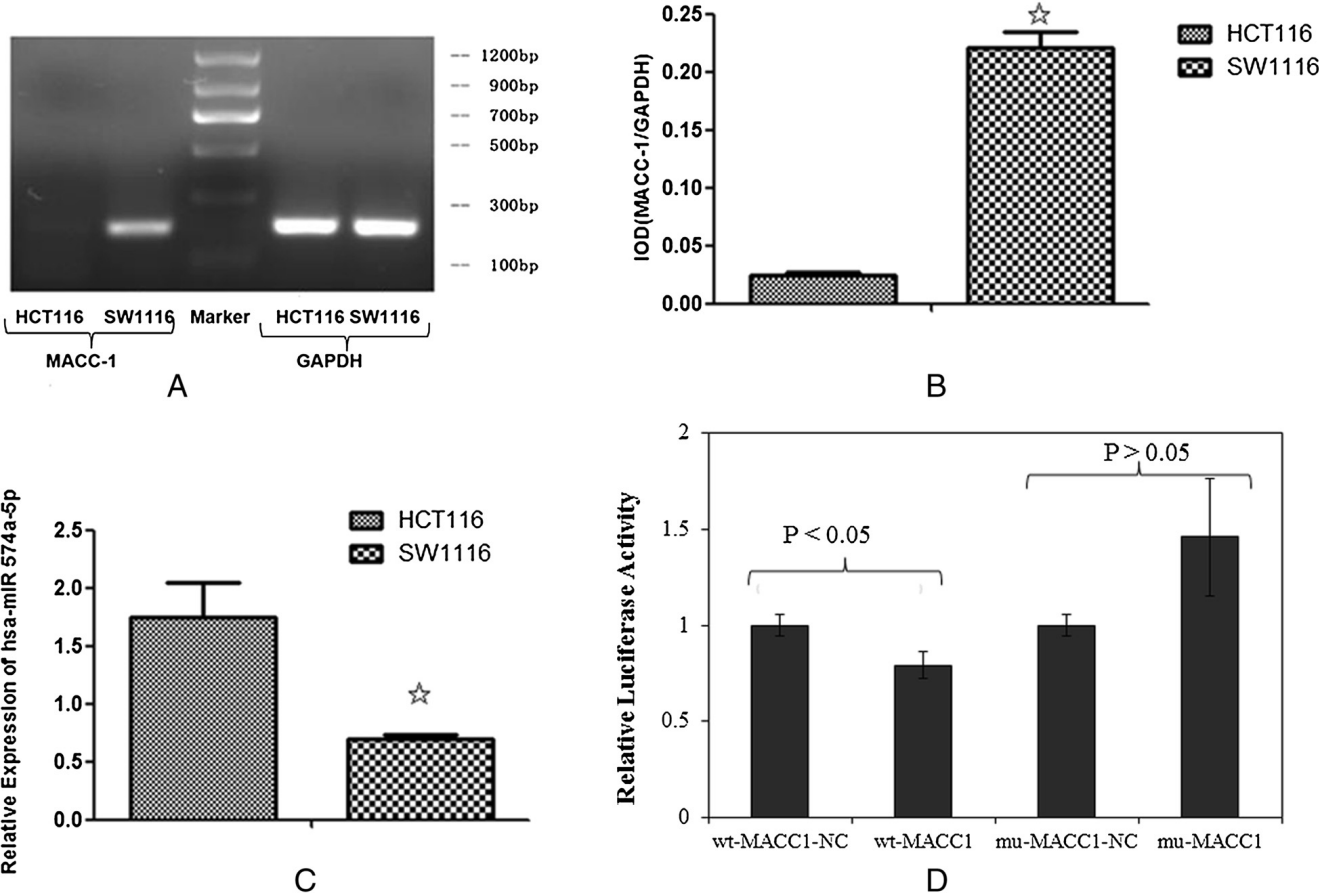
**The mRNA expression levels of MACC-1 and hsa-miR-574-5p in SW1116 and HCT116 cell lines. A&B**. MACC-1 mRNA level in HCT116 cells is significant lower than that in SW1116 cells (^✩^P < 0.01 *vs.* HCT116); **C**. hsa-miR-574-5p mRNA level in HCT116 is significant higher than that in SW1116 cell (^✩^P < 0.01 *vs.* SW1116). **D**. Relative luciferase activity after transfecting has-miR -574-5p into wild type MACC-1 and mutant MACC-1. NC: negative control.

We performed qRT-PCR analysis to detect the mRNA level of MACC-1 and hsa-miR-574-5p in SW1116 and HCT116 cell lines. MACC-1 mRNA level in HCT116 cells with lower metastasis potential was significantly lower than that in SW1116 cells, which has higher metastasis potential (P < 0.01, Figure 2A, B). The mRNA level of hsa-miR-574-5p in HCT116 cells was higher than that in SW1116 cells (P < 0.01, Figure 2C). Our results indicated that an inverse correlation between hsa-miR-574-5p and MACC-1 at mRNA level was observed in the SW1116 and HCT116 cell lines.

### Has-miR-574-5p directly targets MACC-1

We co-transfected MACC-1 (3′UTR sequence) vector and hsa-miR-574-5p or negative control into cells. The luciferase activity of wt-MACC1 group was significantly reduced comparing with wt-MACC1-NC group (P < 0.05, Figure 2D). There were no significant differences between mu-MACC1-NC group and mu-MACC1 group (P > 0.05, Figure 2D), demonstrating that miRNA could not combine to the mutant 3′UTR. The results showed that has-miR-574-5p might inhibit MACC1 expression.

### The effect of hsa-miR-574-5p antisense and miRNA mimics transfection on MACC-1 protein expression in SW1116 and HCT116 cell lines

**Figure 3:**
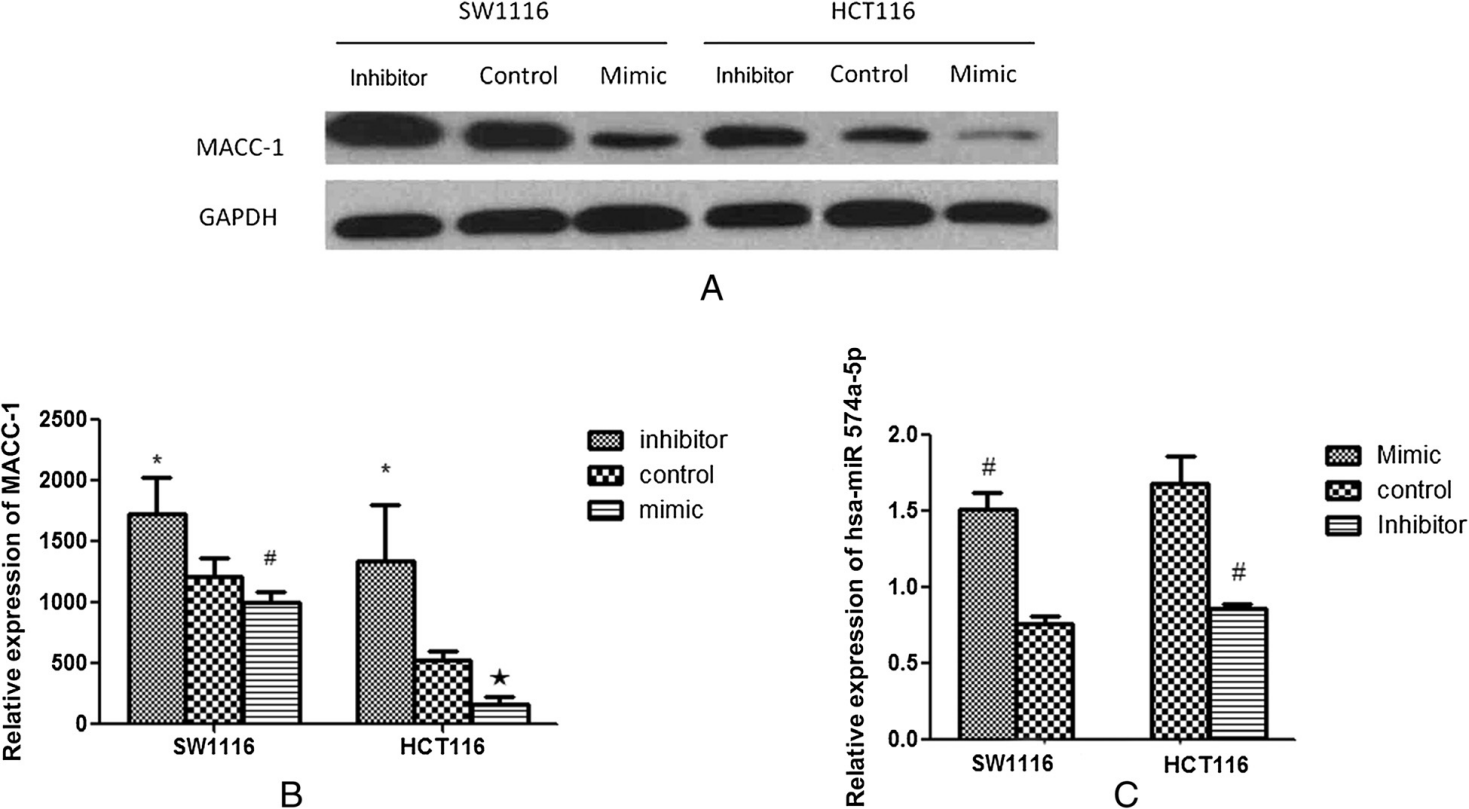
**The effect of hsa-miR-574-5p antisense and miRNA mimics transfection on MACC-1 expression in SW1116 and HCT116 cell lines. A&B**. Effect of hsa-miR-574-5p on MACC-1 expression in HCT116 and SW1116 cells; compared with control group, mimic group reduced MACC-1 expression, while inhibitor group increased MACC-1 expression (*P < 0.05, inhibitor *vs.* control group; ^#^P < 0.05, mimic *vs.* control group; ^★^P < 0.01, mimic *vs.* control group); **C**. hsa-miR-574-5p expression was reduced in antisense (inhibitor group) transfected HCT116 cells, while it was increased in miRNA mimics (mimic group) transfected SW1116 cells (^#^P < 0.01 *vs.* control group).

Compared to corresponding control group, the protein expression of MACC-1 was down-regulated in mimic groups in both SW1116 and HCT116 cell lines (P < 0.05), as shown in Figure 3A, B. Compared to corresponding control group, the protein expression of MACC-1 was up-regulated in inhibitor group in both SW1116 and HCT116 cell lines (P < 0.05), also shown in Figure 3A, B. The protein expression of has-miR-574-5p was reduced in antisense transfected HCT116 cells, while it was increased in miRNA mimics transfected SW1116 cells (Figure 3C). Thus, it was indicated that hsa-miR-574-5p negatively regulated MACC-1 expression at protein level.

### The effect of hsa-miR-574-5p on colony formation, cell invasion and cell spheroid formation in SW1116 and HCT116 cell lines

**Figure 4:**
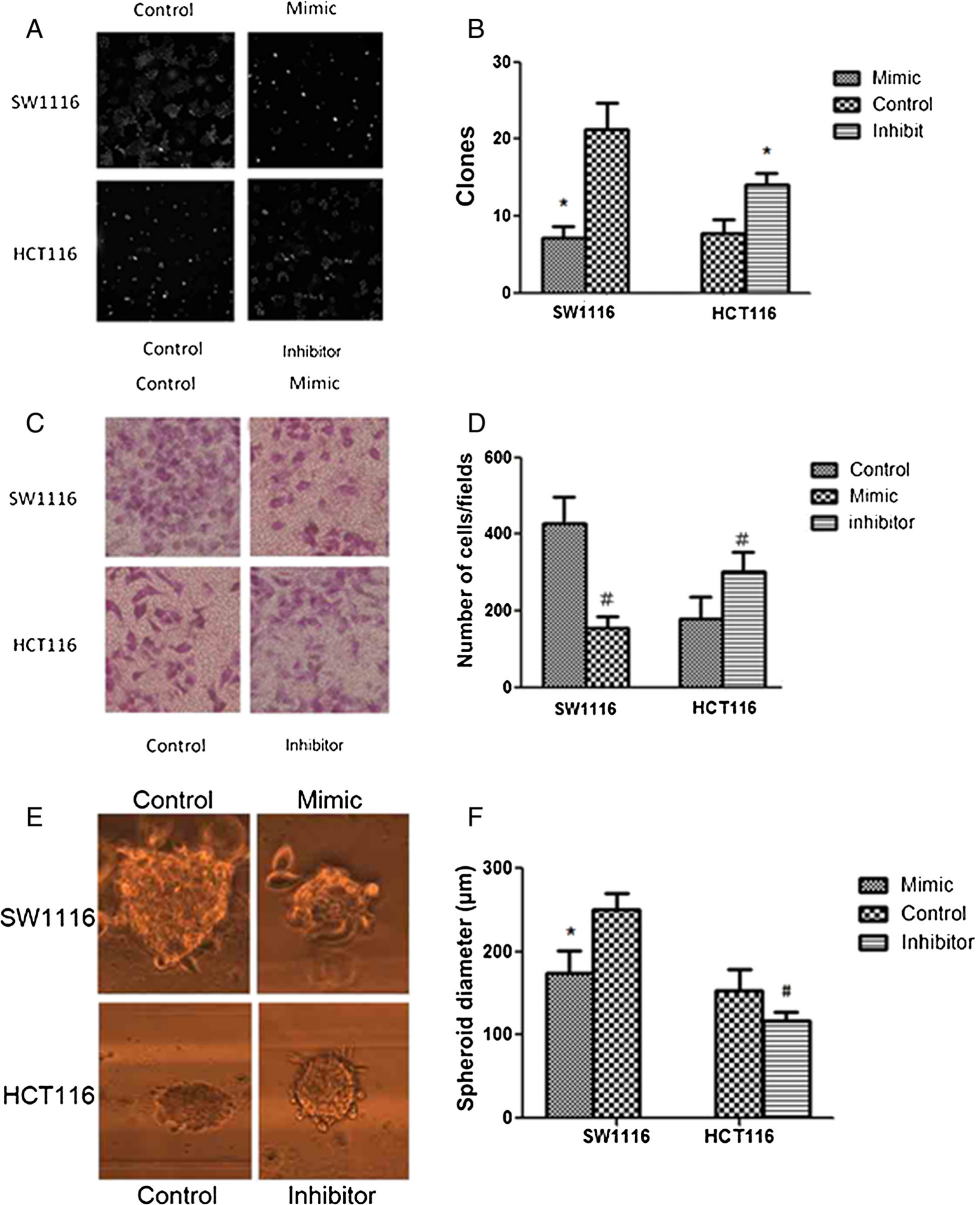
**The effect of hsa-miR-574-5p antisense and miRNA mimics transfection on cell colony formation, cell invasion and cell spheroid formation in SW1116 and HCT116 cell lines. A&B**. The effect of hsa-miR-574-5p antisense and miRNA mimics transfection on cell colony formation in SW1116 and HCT116 cell lines (*P < 0.01 vs. control group). **C&D**. The effect of hsa-miR-574-5p antisense and miRNA mimics transfection on cell invasion in SW1116 and HCT116 cell lines (#P < 0.01 vs. control group); **E&F**. The effect of hsa-miR-574-5p antisense and miRNA mimics transfection on cell spheroid formation in SW1116 and HCT116 cell lines (*P < 0.01 vs. control group, ^#^P < 0.05 vs. control group).

To study the effect of has-miR-574-5p on colony formation, cell invasion and cell spheroid formation, the antisense transfected HCT116 cells and miRNA mimics transfected SW1116 cells were investigated. It showed that the decreased expression of has-miR-574-5p in HCT116 cells stimulated cell proliferation and invasive activity, inducing increased colony formation, cell invasion and cell spheroid formation of HCT116 cells, compared to control group (P < 0.05), as shown in Figure 4. Conversely, the increased expression of has-miR-574-5p in SW1116 cells decreased colony formation, cell invasion and cell spheroid formation, compared to control group (P < 0.05), as shown in Figure 4.

## Discussion

Lots of reports have shown that miRNAs are important regulators for functional genes involved in oncogenesis and development. The miRNAs abnormalities are found in various key processes of tumor metastasis, in which miRNAs can regulate a variety of genes and are pivotal for invasion or metastasis [14,24-29]. In the present work, we identified the miRNAs profile that might regulate the MACC-1 gene expression by bioinformatics analysis. The hsa-miR-574-5p was chosen for further analysis. Subsequently, results demonstrated that hsa-miR-574-5p was involved in the regulation of MACC-1, playing a functional role in colorectal cancer liver metastasis.

We successfully constructed liver-metastatic nude mice model by injecting two human colorectal cancer cell lines labeled with GFP through spleen. SW1116 cells represented a higher metastasis potential in the model than that of HCT116 cells, so we selected these two cell lines for further function analysis. The E value of hsa-miR-574-5p is relatively lower. Inhibition of hsa-miR-574-5p suppressed the growth of colorectal tumors in the nude mice [22]. So the hsa-miR-574-5p is chosen for investigating if this miRNA is correlated with the expression of MACC-1.

In this study, qRT-PCR analysis indicated that an inverse correlation between hsa-miR-574-5p and MACC-1 at mRNA level was observed in the SW1116 and HCT116 cell lines. Luciferase experiments showed that miRNA could bind to the 3′UTR but not the mutant 3′UTR. Furthermore, the effect of hsa-miR-574-5p antisense and miRNA mimics transfection on MACC-1 expression in SW1116 and HCT116 cell lines showed that hsa-miR-574-5p negatively regulated MACC-1 expression at protein level. Therefore, it was verified that hsa-miR-574-5p was negatively involved in the regulation of MACC-1 at mRNA and protein levels. Subsequently, we investigated the functional role of hsa-miR-574-5p in the colorectal cancer liver metastasis. Though HCT116 cells represented a low metastasis potential, siRNA mediated knockdown of has-miR-574-5p increased colony formation, cell invasion and cell spheroid formation in HCT116 cells, compared to control group. The reverse result was obtained in the miRNA mimics transfected SW1116 cells. It was hypothesized that hsa-miR-574-5p affected colony formation, cell invasion and cell spheroid formation by mediating the expression of MACC-1.

Increased MACC-1 expression in colorectal cancer cells can induce proliferation, migration and invasion of cancer cells in vitro, while promoting liver metastasis in a xenograft model [30]. Previous research also showed that MACC-1 protein in colorectal cancer could bind to c-met promoter through nuclear translocation to up-regulate c-met gene transcription and expression [9,31-33]. The activation of HGF/c-Met signaling pathway not only can promote the spread of colorectal cancer cells [9], but also can promote colorectal cancer liver metastasis [34]. In contrast, the inhibition of this pathway can reduce tumor invasion and metastasis [35]. Therefore, we speculated that hsa-miR-574-5p played a suppressive role in colorectal cancer liver metastasis by negatively involved in the down-regulation of MACC-1 expression.

It was verified that hsa-miR-574-5p was negatively involved in the regulation of MACC-1 at mRNA and protein levels. Moreover, hsa-miR-574-5p affected the colony formation, cell invasion and cell spheroid formation. Taken together, our findings partly elucidated that hsa-miR-574-5p played a suppressive role in colorectal cancer liver metastasis by negatively directing the expression of MACC-1. The results in this study might offer a novel therapy option of hsa-miR-574-5p in colorectal cancer liver metastasis. However, there are some limitations in this study. First, we just only investigated the hsa-miR-574-5p function of invasion in vitro. It is needed to demonstrate the role by in vivo experiments. Second, although the hsa-miR-574-5p may be the potential therapy for colorectal cancer liver metastasis therapy, the function of it in patients with colorectal cancer liver metastasis need further experiments to explore.

## Abbreviations

EMT: Epithelial-mesenchymal transition; CSC: Cancer stem cells; MACC-1: Metastasis-associated in colon cancer 1; GFP: Green fluorescent protein; GAPDH: Glyceraldehyde-3-phosphate dehydrogenase.

## Competing interests

The authors declare that they have no competing interests.

## Authors’ contributions

ZC carried out the molecular genetic studies, participated in the sequence alignment and drafted the manuscript. JT carried out the immunoassaysand participated in the sequence alignment. JC participated in the design of the study and performed the statistical analysis. ZW conceived of the study, and participated in its design and coordination and helped to draft the manuscript. All authors read and approved the final manuscript.
